# Transcriptome divergence during leaf development in two contrasting switchgrass (*Panicum virgatum* L.) cultivars

**DOI:** 10.1371/journal.pone.0222080

**Published:** 2019-09-12

**Authors:** Nathan A. Palmer, R. V. Chowda-Reddy, Anthony A. Muhle, Satyanarayana Tatineni, Gary Yuen, Serge J. Edmé, Robert B. Mitchell, Gautam Sarath

**Affiliations:** 1 Wheat, Sorghum, and Forage Research Unit, USDA-ARS, Lincoln, Nebraska, United states of America; 2 Department of Plant Pathology, University of Nebraska-Lincoln, Lincoln, Nebraska, United states of America; Universidade de Lisboa Instituto Superior de Agronomia, PORTUGAL

## Abstract

The genetics and responses to biotic stressors of tetraploid switchgrass (*Panicum virgatum* L.) lowland cultivar ‘Kanlow’ and upland cultivar Summer are distinct and can be exploited for trait improvement. In general, there is a paucity of data on the basal differences in transcription across tissue developmental times for switchgrass cultivars. Here, the changes in basal and temporal expression of genes related to leaf functions were evaluated for greenhouse grown ‘Kanlow’, and ‘Summer’ plants. Three biological replicates of the 4^th^ leaf pooled from 15 plants per replicate were harvested at regular intervals beginning from leaf emergence through senescence. Increases and decreases in leaf chlorophyll and N content were similar for both cultivars. Likewise, multidimensional scaling (MDS) analysis indicated both cultivar-independent and cultivar-specific gene expression. Cultivar-independent genes and gene-networks included those associated with leaf function, such as growth/senescence, carbon/nitrogen assimilation, photosynthesis, chlorophyll biosynthesis, and chlorophyll degradation. However, many genes encoding nucleotide-binding leucine rich repeat (NB-LRRs) proteins and wall-bound kinases associated with detecting and responding to environmental signals were differentially expressed. Several of these belonged to unique cultivar-specific gene co-expression networks. Analysis of genomic resequencing data provided several examples of NB-LRRs genes that were not expressed and/or apparently absent in the genomes of Summer plants. It is plausible that cultivar (ecotype)-specific genes and gene-networks could be one of the drivers for the documented differences in responses to leaf-borne pathogens between these two cultivars. Incorporating broad resistance to plant pathogens in elite switchgrass germplasm could improve sustainability of biomass production under low-input conditions.

## Introduction

Switchgrass (*Panicum virgatum* L.) is a native, perennial, warm-season grass with a documented range extending from Central America to Canada [1]. Two ecotypes of switchgrass are known, with the lowland ecotypes adapted to wetter regions, and the upland ecotypes adapted to drier conditions. Upland and lowland cultivars of switchgrass differ in their genetics [2–7], yield potential [8–13], upland cultivars are better adapted for cold, winter survival [14–16], and lowland ecotypes exhibit higher resistance to certain pathogens [17–19].

Switchgrass is a model biomass/biofuel crop that is frequently utilized as a source of forage and for conservation [1]. More specifically, as a source of biomass, switchgrass is targeted to be cultivated with minimal external inputs on marginal soils that are not best suited for row crops [8, 20, 21]. Sustainable production of biomass from switchgrass is dependent on stable yields over several years and good local adaptation of the cultivars within their regions of production. Because infection by viral pathogens can drastically suppress growth in susceptible switchgrasses [19] and significant losses in ethanol yields can result from severe infestations by rust fungi (*Puccinia emaculata*) [22], there is uncertainty of yield stability of switchgrass under disease pressures.

Plants contain several mechanisms for monitoring the environment, with several specifically evolved for detecting pathogens [23]. Genes encoding nucleotide-binding leucine rich repeat proteins (NB-LRR) comprise the largest numbers of disease resistance genes (*R* genes) known in plants [24–27]. *R* genes are usually part of large gene families with many expressed at low levels in plant cells, suggestive of their role in monitoring [28]. Because NB-LRRs can participate in protein-protein interactions and are present in large complexes, it is likely they recognize changes in host proteins that respond to pathogen elicitors and subsequently catalyze downstream reactions [28–30]. Once a NB-LRR-catalyzed signaling cascade is triggered, it results in a significant redirection of plant metabolism with a range of resistance responses [31]. Although several of these resistance-related processes have been investigated in other plants, there is considerable lack of data in switchgrass and related perennial feedstocks. Frazier et al. [32] identified the NB-LRR genes in the switchgrass genome (version1.1) and catalogued their expression profiles in diverse switchgrass germplasm. Among the findings consistent with data presented by Uppalapati et al. [18], fungal-resistant plants appeared to contain greater transcript abundances for specific NB-LRR genes than susceptible plants in the absence of a pathogen, potentially due to enhanced monitoring for infection in such plants.

Several other classes of genes and the proteins they encode, such as wall-bound kinases, NADPH-oxidases, protein kinases, protein phosphatases, and transcription factors also participate in monitoring environmental changes in plants either directly or indirectly via interactions with primary signals [33–35]. Among transcription factors, genes encoding WRKYs respond strongly to environmental stress, and play a significant role in plant immunity [36]. Switchgrass contains at least 240 WRKY genes [37]. Several of these WRKYs were associated with specific developmental stages of flag leaves in field-grown switchgrass, and 23 WRKY genes were associated with a senescence-associated gene co-expression module [37].

An RNA-Seq study of flag leaf development in field-grown switchgrass developed a framework to understand the molecular signatures associated with development through senescence onset for switchgrass [38]. Other similar studies have resulted in the identification of specific NAC transcription factors that can positively modulate leaf senescence in switchgrass [39, 40]. Upland and lowland leaf transcriptomes have also been analyzed by RNA-Seq [41], although these authors only analyzed a single leaf from one individual genotype of three cultivars at one harvest date, making developmental and upland versus lowland comparisons difficult. However, their data indicated that transcripts associated with photosynthesis and cellular components related to photosynthesis were significantly more abundant in the lowland leaves relative to the single upland leaf analyzed. Additionally, several genes potentially involved with plant defense, such as those encoding catalase, S-adenosylmethionine synthase, and wound-induced protein were differentially regulated in the upland leaf sample as compared with the lowland leaf samples. How representative these changes were across genotypes or development were not explored.

Crosses between a tetraploid upland switchgrass cultivar ‘Summer’ (♀) and a tetraploid lowland cultivar ‘Kanlow’ (♂) were heterotic for yield [10, 11]. Stabilized half-sib families arising from such hybrids outperformed the maternal Summer population for yield and outperformed the paternal Kanlow population for winter survival. Recurrent selection from many half-sib families led to the release of a first bioenergy-type switchgrass cultivar “Liberty” that was adapted to the US Central Great Plains [42]. However, several Summer x Kanlow hybrid switchgrass plants, including the cultivar Liberty, suffered from increased disease pressure as compared to the Kanlow parent [19], suggesting that potential disease-related traits in Kanlow were not yet incorporated into hybrid progeny. Moreover, there are limited data on the genes related to surveillance mechanisms and pathogen defense in Kanlow.

This study was undertaken to determine the temporal changes in leaf gene expression in Kanlow and Summer plants grown under controlled (greenhouse) conditions. The goals were to: (1) develop a foundational dataset of the gene co-expression networks that impact overall leaf functions; (2) discover similarities and differences in NB-LRR and receptor-like kinase (RLK) genes associated with surveillance and defense in Kanlow and Summer plants; (3) distinguish co-expression modules that were shared or unique to either cultivar; (4) identify some NB-LRR genes with population-specific transcription.

## Materials and methods

### Plant materials

Seeds of the two tetraploid switchgrass cultivars, Kanlow and Summer, were obtained from plants grown in field nurseries. Nurseries were planted and maintained in fields belonging to the University of Nebraska Agricultural Research Division, near Ithaca, NE [43]. Both of these cultivars are synthetic populations derived from a collection of lowland and upland plants [44].

### Plant growth conditions and harvests

Plants were raised from seeds planted in cone-tainers (3.8 cm diameter by 21 cm deep;

Ray Leach SC10; Stuewe & Sons, Inc, Tangent, OR) containing a soil mixture composed of 4:4:1.5:0.5 parts peatmoss:vermiculite:sand:topsoil with added micronutrients, wetting agent, pH control (lime) and iron. Prior to planting, cone-tainers were well watered and lightly tapped to settle soil and remove air gaps. Several seeds were distributed on top of the wetted soil mixture, lightly covered with a layer of the dry soil mixture and gently watered. Two weeks following germination, plants were thinned to leave one seedling per cone-tainer. At this time, cone-tainer racks were moved to large plastic tubs to facilitate water absorption through the soil. Plants were maintained in a greenhouse with a 16h day, 27 ± 3°C, 8h night 22 ± 3°C cycle. Supplemental lights were provided by a bank of LEDs (Lumigrow Pro 650E, 1100 μmol s^-1^, Lumigrow Inc, Emeryville, CA). Approximately 350 plants were tagged upon the emergence of the third leaf on the primary tiller to provide adequate plants at similar leaf developmental stages for the duration of sampling.

Sampling was confined to the 4^th^ leaf on the primary tiller, with new plants used at each sampling date. Plants were discarded once the 4^th^ leaf was excised. Leaves were collected following emergence until late visible senescence for a total of seven harvests that occurred over a 60 day period. The first sampling date (D0) was at the emergence of the 4^th^ leaf, successive harvests occurred at approximately 7–13 day intervals. Leaves were collected from three replicate samples for a total of 42 samples (2 populations x 7 harvest dates x 3 replicates), each containing 15 randomly selected individual plants per replicate to maximize population-specific differences and minimize genotype-specific effects. The 15 excised leaves per replicate were pooled, cut into approximately 6 to 8 cm pieces, placed within 50 mL polypropylene tubes, capped, and flash frozen with liquid N_2_. Frozen samples were placed on dry ice for transport to the laboratory and subsequently stored at– 80 °C until needed.

### Plant analyses

All samples were cryogenically ground with liquid N_2_ using mortars and pestles. Ground leaf material was stored at -80 °C. Aliquots of approximately 50 mg of ground material were used for total chlorophyll measurements [38]. For total N analyses, approximately 300 mg of ground leaf samples were transferred to borosilicate glass tubes (0.5 cm x 8 cm) and dried at 50 °C in a forced air oven. Aliquots of approximately 10 mg of oven-dried samples were analyzed for total N [45].

### RNA extraction, library preparation and sequencing

Total RNA was extracted from approximately 100 ± 10 mg aliquots as described earlier [43] and used for 3’sequencing. As compared to traditional Illumina TruSeq libraries, 3’ libraries provide a cost effective strategy for monitoring transcriptomes, since many more libraries can be sequenced on a single flowcell of a high-throughput sequencer (https://www.lexogen.com). 3’-sequencing has been used recently in plant research [46, 47].

Aliquots of total RNA were submitted to the University of Nebraska Medical Center Genomics Core Facility, Omaha, NE (www.unmc.edu/vcr/cores/vcrcores/dna-sequencing) for all further analyses. Briefly, 500 ng of total RNA was processed according to manufacturer’s supplied protocol for 3’-library generation (Lexogen QuantSeq 3’ mRNA-Seq Library Prep Kit FWD for Illumina, Lexogen GmbH, Vienna, Austria), with a PCR amplification for 14 cycles. RNA and libraries were checked for quality using Qubit (ThermoFisher Scientific, Waltham, MA) and an Agilent 2100 Bioanalyzer (Agilent Technologies, Santa Clara, CA). Forty-two individual libraries were pooled with a loading concentration of 1.3pM and sequenced on NextSeq500 (Illumina, Inc., San Diego, CA), using a single high output flowcell and sequencing kit to obtain 75-bp single reads. Run quality was monitored using Basespace (Illumina, Inc., San Diego, CA). Raw reads for all 42 samples can be found under NCBI BioProject PRJNA528942.

### Bioinformatic analyses

Demultiplexed raw reads were trimmed using bbduk, part of BBTools (https://jgi.doe.gov/data-and-tools/bbtools/), with the following parameters: k = 13, ktrim = r, useshortkmers = t, qtrim = r, trimq = 10, minlength = 20, mink = 5, ref = polyA.fa.gz,truseq_rna.fa.gz. Trimmed reads were then aligned to version 4.1 of the switchgrass genome (https://phytozome.jgi.doe.gov) using hisat2 [48]. Samtools was used to convert alignments to sorted BAM files [49] and gene expression counts were calculated for reads uniquely mapped to exons using featureCounts [50].

Multi-dimensional scaling (MDS) plots were generated using the metaMDS function in the vegan package [51] in R [52] with Euclidean distance measures. Prior to differential expression analysis, lowly-expressed genes were removed from the dataset by requiring each gene to have more than two counts per million (CPM) in at least three of the 42 total samples. Differential expression analysis was done using the DESeq2 package in R [53], with significance thresholds of false discovery rate < = 0.05 and log_2_ fold change > 1.0. Signed co-expression networks were generated using the CPM filtered genes normalized using the varianceStabilizingTransformation function in DESeq2 and WGCNA package in R [54] with the following parameters: power = 14, minClusterSize = 30, and cutHeight = 0.25. The GeneOverlap package [55] in R was used to analyze KEGG pathway, KO, and PFAM enrichment in the co-expression modules using a Fisher’s exact test approach.

Genomic resequencing data for Kanlow (NCBI BioProject PRJNA265642) and Summer (NCBI BioProject PRJNA258732) were aligned to version 4.1 of the switchgrass genome using bowtie2 (version 2.3.1) [56] using—sensitive,—fr, and the remaining parameters at default settings. Read pairs mapped to gene coding regions were counted using featureCounts [50] and used for calculating genomic coverage for each gene in the Kanlow and Summer populations. The software IGV was used for genomic coverage visualization [57, 58].

Putative receptor-like kinases (RLKs) were identified in the switchgrass genome using hmmsearch (version 3.1; www.hmmer.org) and the Hidden-Markov Models for classifying all plant kinases generated by Lehti-Shiu and Shui [59]. Loci encoding proteins in the RLK/Pelle family were considered as putative RLKs.

Due to the relatively small sample size and lack of complexity, chlorophyll and N data were analyzed using ANOVA in Excel, followed by Tukey’s Honestly Significant Difference (HSD) post-hoc analysis using *P* ≤0.05 as a cutoff for significance.

## Results

### Chlorophyll and total nitrogen content increase and decrease similarly in both cultivars

**Fig 1:**
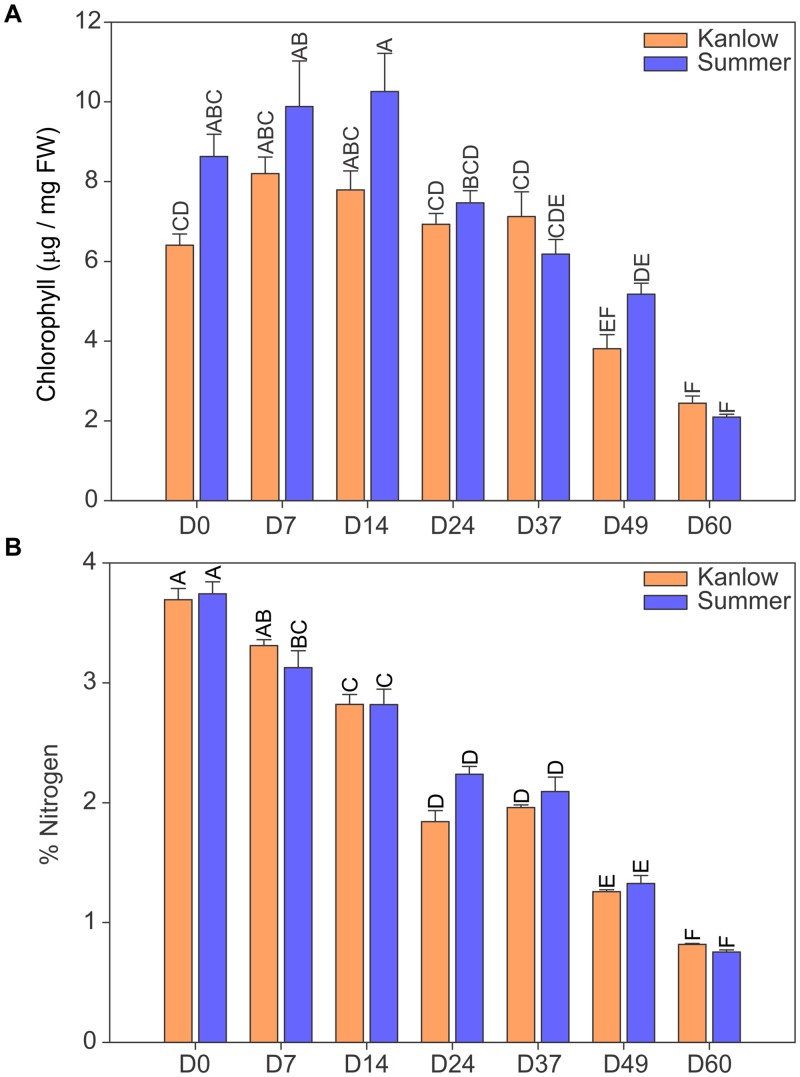
Changes in leaf components over time. (A) chlorophyll and (B) total N. The 4^th^ leaf was harvested from upland Summer and lowland Kanlow switchgrass cultivars starting at emergence (D0) followed by six successive harvests until late senescence (D60). Summer (blue bars); Kanlow (orange bars). Error bars are ± s.e. Different letters over each bar denote statistical significance at *P* ≤ 0.05.

Chlorophyll content and total N were used as reliable indicators of leaf development and senescence for both cultivars. Chlorophyll content increased between the first and third harvests in Summer leaves and decreased significantly thereafter (Fig 1A). In contrast, leaf chlorophyll content increased for the D7 harvests in Kanlow as compared to the D0 harvest, did not change significantly for ~30 days, and declined significantly thereafter (Fig 1A). Although peak chlorophyll content was higher in Summer leaves it was not significantly different than peak chlorophyll content in Kanlow leaves.

Changes in total leaf N content were similar for both cultivars, where peak N content was observed in emerging leaves, D0 harvests. Total leaf N decreased subsequently at each successive harvest with lowest total leaf N observed at the last D60 harvest (Fig 1B).

### Mapping reads onto the switchgrass genome

Overall, there was an average of 10.2M filtered reads per sample with an average length of 61 bases and average quality score of 34.5. There was an average mapping rate of 85% (74% uniquely-mapped and 11% multi-mapped) to version 4.1 of the switchgrass genome with 68.75% uniquely mapped to and counted in annotated gene exons. However, a significant difference in mapping rates was observed between the Kanlow and Summer populations with Kanlow having higher mapping (88.6% vs 81.5%) and counted (71.5% vs 66%) rates than Summer. This difference is most likely due to the fact that the reference genome is from a lowland ecotype (‘Alamo’), like Kanlow, while Summer is an upland ecotype.

### Leaf transcriptomes follow similar profile trends but are distinguished by MDS plots

**Fig 2:**
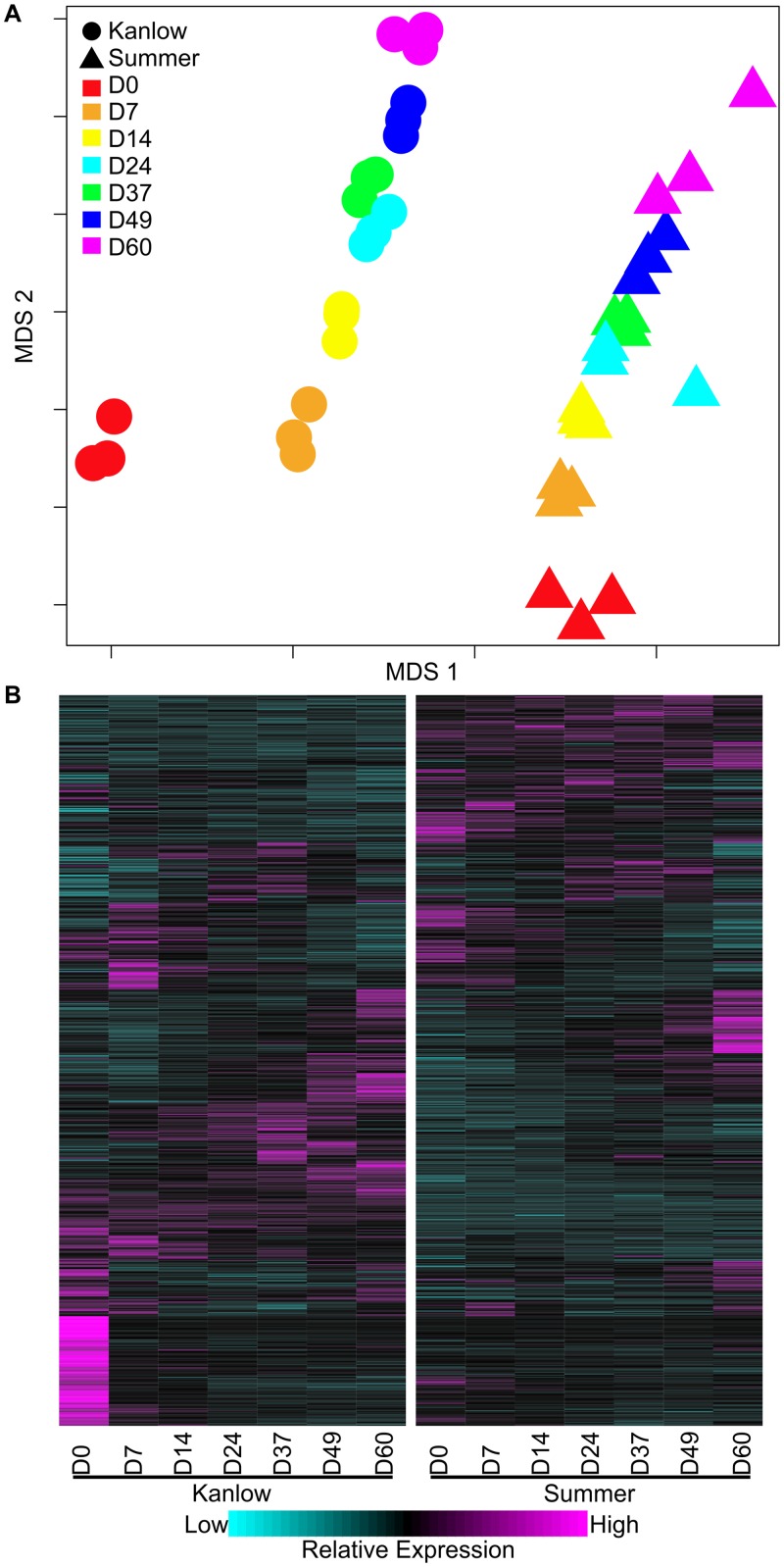
Global leaf transcriptome analysis. (A) MDS analysis and (B) gene expression heatmap. Harvest dates as described for Fig 1. In panel A, Kanlow transcriptomes are in circles and Summer transcriptomes are in triangles. Each harvest date is color coded. In panel B, one way clustering was performed and the dendrograms removed for ease of presentation. Expression profiles are shown as z-scores with magenta = high expression and cyan = low/no expression.

Leaf transcriptomes were subjected to MDS analysis. Transcriptomes of Summer leaves were distinguished from those of Kanlow leaves in the first dimension (Fig 2A), but the overall change in transcriptome profiles across leaf development was similar in the second dimension. Curiously, the Kanlow D0 transcriptomes (red circles, Fig 2A) were distinguished from all the other transcriptomes. These similarities and differences in transcriptome profiles suggested both cultivar-specific and cultivar-independent gene expression as potential underlying causes.

To further explore these nuances in gene-expression profiles, heatmaps of differentially expressed genes (DEGs) were constructed to visualize global gene expression profiles (Fig 2B). Emerging Kanlow 4^th^ leaves (D0) contained a cluster of highly expressed genes that were expressed in much lower levels across all the other time points in both cultivars. At all subsequent time points, individual harvest date comparisons of transcriptomes more likely reflected basal ecotype and time-dependent driven differences in expression. Analysis of DEGs with or without the Kanlow D0 datasets did not appreciably affect these overall findings, suggesting that the Kanlow D0 transcriptomes were part of normal leaf development. Small discrepancies in leaf emergence and developmental stages between Summer and Kanlow plants at the D0 sampling point could have accounted for these differences.

### Genes encoding enzymes for important leaf metabolic processes follow a similar trend in both ecotypes

**Fig 3:**
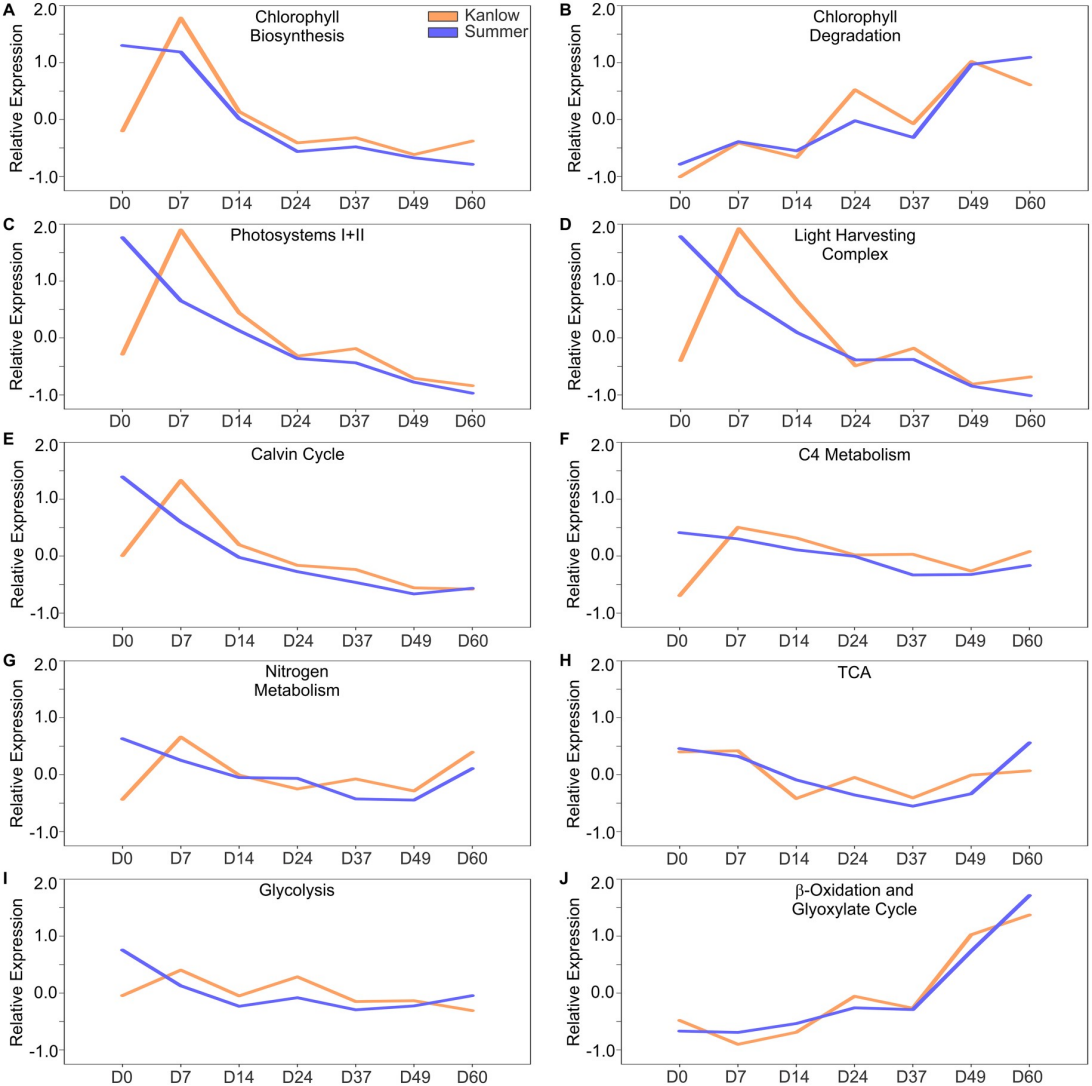
Expression of profiles of genes associated with central aspects of leaf function and metabolism. (A) chlorophyll biosynthesis, (B) chlorophyll degradation, (C) photosystems, (D) light harvesting complex, (E) Calvin cycle, (F) C4-metabolism, (G) nitrogen metabolism (H) TCA, (I) glycolysis, and (J) β-oxidation and glyoxylate cycle in Kanlow (orange lines) and Summer (blue lines). Both differentially expressed and non-differentially expressed transcripts from genes with at least 100 normalized counts at one time point were included. Paralogous genes with low counts were excluded in these analyses. Counts for individual transcripts were converted to z-scores and averaged for each time point.

The gene expression profiles of metabolic processes central to leaf function were evaluated for the two contrasting cultivars by summing the transcript counts of paralogous genes with the same protein annotation (for example: PEP carboxylase, glutamine synthetase, etc.,) and predicted functional pathway (for example: Chlorophyll biosynthesis, Calvin cycle, etc.,) with expression across harvest dates. With minor variations (outside of the D0 Kanlow transcriptomes) all these processes followed a similar trend (Fig 3). Genes encoding proteins required for chlorophyll biosynthesis were elevated at the first two harvests in Summer and the second harvest in Kanlow (Fig 3A), and thereafter declined in parallel in both cultivars. Similarly, expression of genes associated with chlorophyll degradation increased over time, with a jump in expression recorded between the D37 and D49 harvest dates, probably coincident with the onset of leaf senescence (Fig 3B). Likewise, expression of genes required for photosystems (Fig 3C), light harvesting complexes (Fig 3D), and the Calvin cycle (Fig 3E) mirrored the trend seen for expression of genes associated with chlorophyll biosynthesis (Fig 3A).

Outside of the D0 transcriptomes of Kanlow leaves, expression of genes encoding enzymes of the C4 metabolism (Fig 3F), nitrogen metabolism (Fig 3G), TCA cycle (Fig 3H), and glycolysis (Fig 3I) were relatively unchanged, although there was a modest increase in overall expression of genes associated with TCA in Kanlow (D60 vs D49; Fig 3H) and in nitrogen metabolism for both cultivars (Fig 3G).

There was strong upregulation of genes that form part of the β-oxidation and glyoxylate cycle as leaves aged. Highest expression of these genes was seen at the last two harvest dates (D49 and D60; Fig 3J), when leaves were visibly senesced and had lost significant amounts of chlorophyll (Fig 1A). The identities of the genes associated with the pathways described in Fig 3 are given in S1 Data.

### Leaf senescence is accompanied by the upregulation of a family of NAC transcription factors and senescence associated genes in both cultivars

**Fig 4:**
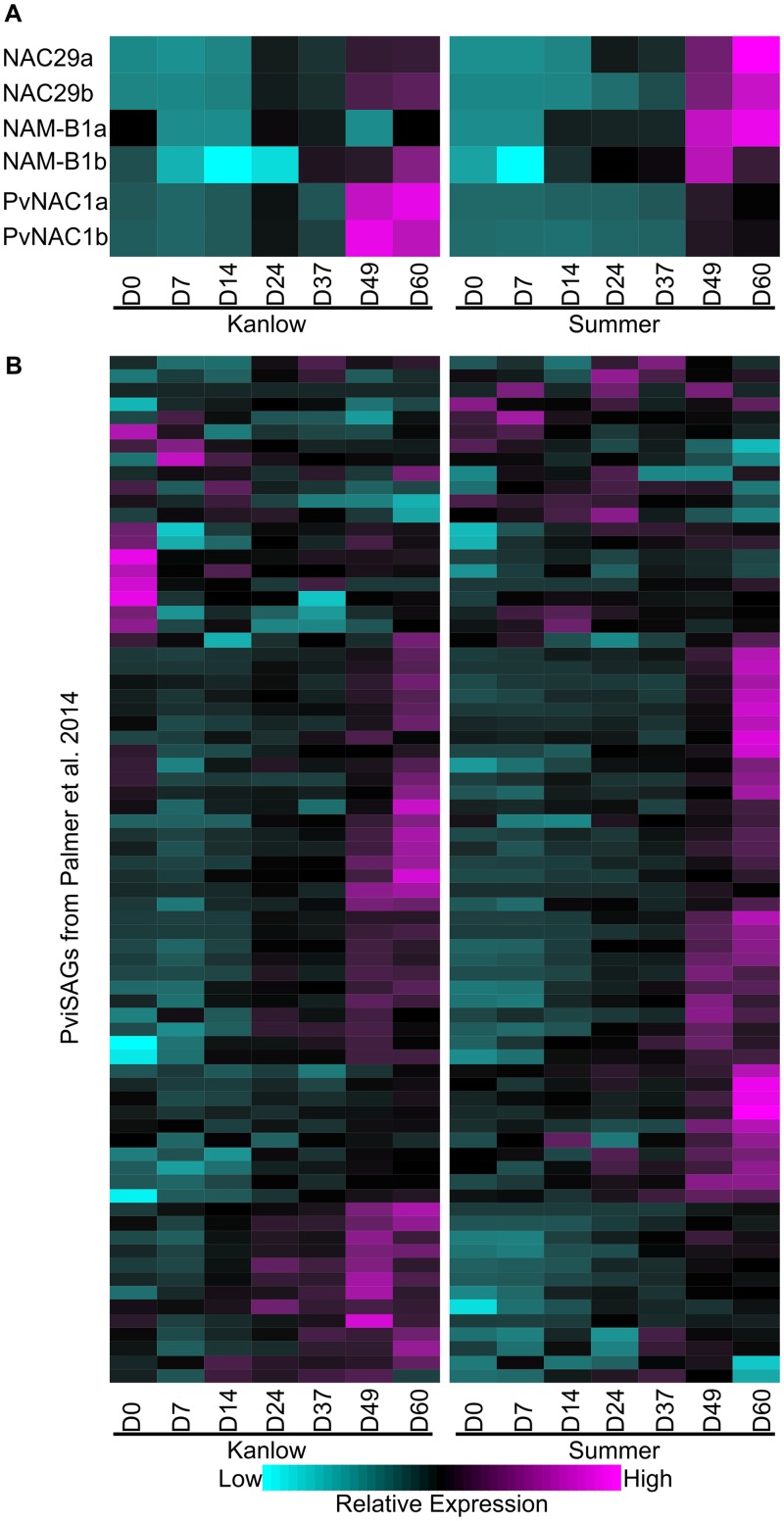
Expression profiles of select leaf senescence-associate genes. (A) Three NAC genes and homeologs, (B) Switchgrass homologs of Arabidopsis senescence-associated genes (SAGs). For heat maps, one way clustering was performed and the dendrograms removed for ease of presentation. Expression profiles are shown as z-scores with magenta = high expression and cyan = low/no expression. Gene identities are provided in S1 Data.

Previous work had established the association of several transcription factors including NACs (NAC29 and NAMB1) with flag leaf senescence in the upland cultivar Summer [38] and NAC1 with the lowland cultivar Alamo [39, 40]. Additionally, there was a significant temporal upregulation of many switchgrass homologs of Arabidopsis senescence-associated genes (SAGs) during flag leaf senescence [38]. Using these previous datasets as guides, the expression profiles of specific NACs and SAGs were evaluated (Fig 4).

NAC genes, namely NAC29, NAMB1, and NAC1, were upregulated in aging switchgrass leaves and transcripts for these genes were most abundant in leaves from both cultivars at the last two harvest dates (Fig 4A**)**. Although there were some minor differences in the expression of these NACs between the two cultivars, the overall patterns were similar, suggesting that the timing and progression of senescence were similar in Kanlow and Summer leaves.

The expression profiles of SAGs are shown in Fig 4B. A majority (52 out of 74) of the SAG genes had profiles consistent with association with leaf senescence as they were most highly expressed at the last two harvest dates (D49 and D60; Fig 4B; S1 Data). However, the other 22 switchgrass SAG homologs (S1 Data) did not display an expression pattern consistent with an association with leaf senescence. Whether, this arises because of an imperfect orthology assignment during mapping onto the switchgrass genome or due to cultivar and ecotype differences in gene expression remains unclear.

Similar and dissimilar relative expression of several SAGs were documented between Kanlow and Summer leaves. As examples of genes with similar expression profiles in the two ecotypes were Pavir.3KG204200 (WRKY), Pavir.2KG378800 (STAYGREEN), Pavir.5NG016400, Pavir.5KG008500 (NON-YELLOW COLOR1), and Pavir.1NG278100 (SAG15, EARLY RESPONSIVE TO DEHYDRATION 1), all genes with well-established roles in leaf senescence. Several other SAGs were expressed at a higher level in senescing Kanlow than in senescing Summer leaves: these included Pavir.8NG318800 (TRANSLATIONALLY CONTROLLED TUMOR PROTEIN), Pavir.5NG537600 (TRANSLATION INITIATION FACTOR SUI1 FAMILY PROTEIN), Pavir.3KG137500 (WRKY), Pavir.1NG557000 (PEROXISOMAL 3-KETOACYL-COA THIOLASE 3), Pavir.2NG448400 (STAYGREEN), Pavir.5KG667800 and Pavir.3KG002200 (NAC transcription factors).

Genes that were more abundantly expressed in senescing Summer leaves than in Kanlow leaves included Pavir.3KG511800 (LUMAZINE SYNTHASE), Pavir.5KG515900 (NADP-MALIC ENZYME 3), Pavir.4NG310000 (CATALASE 2), Pavir.1KG308600 (SAG15, EARLY RESPONSIVE TO DEHYDRATION 1), Pavir.9NG180200 (NOL; NON-YELLOW COLOR 1-LIKE), Pavir.9NG090300 (ATP-SULFURYLASE), and Pavir.6NG064300 (ALEURAIN-LIKE PROTEASE) (S1 Data). These data highlight the preferential upregulation of specific loci in the two cultivars and provide some evidence for subtle differences in the execution of the leaf senescence program between Summer and Kanlow plants.

### Network analyses provides evidence for differentially regulated gene expression in the two cultivars

**Fig 5:**
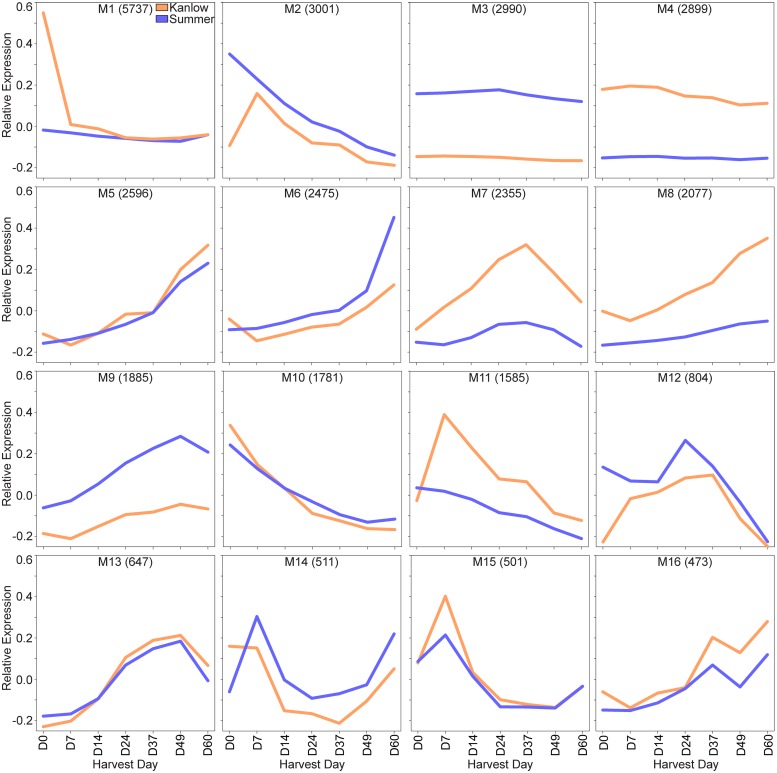
Leaf gene co-expression modules. Kanlow (orange lines) and Summer (blue lines) over the time course of the experiment. In each panel, relative expression (based on module eigengenes) is on the Y-axis and harvest times on the X-axis. Total numbers of genes in each module are indicated in parenthesis.

Gene co-expression network modules (referred to as M in the text) determined by analyses of the RNA-Seq datasets are shown in Fig 5. All other data associated with these analyses are provided in S1 Data. Modules were broadly separated into 3 categories: (1) those in which the two cultivars shared a similar profile of gene expression over time (M5, M6, M10, M13, M14, M15, and M16) or those differing markedly only at the first harvest date (M1, M2 and M12); (2) those that were similar in profile, but genes were more highly expressed in one or the other cultivar (M3, M4, and M9); and (3) those that had somewhat dissimilar overall profiles with expression levels being greater in Kanlow leaves than in Summer leaves (M7, M8 and M11).

Predicted proteins encoded by genes that were part of each co-expression module were queried for their association with the Kyoto Encyclopedia of Genes and Genomes (KEGG) pathways [60]. Enrichment of KEGG pathways was variable across each module, with some modules having greater numbers of significantly enriched pathways (S1 Data). Within M1, 18 KEGG pathways were significantly enriched and this enrichment appears to be driven by the developmental status of the Kanlow 4^th^ leaf at the time of the first harvest. Enriched pathways in M1 included DNA replication, starch and sucrose metabolism, cell wall polymer biosynthesis, cell cycle, RNA polymerase, and base excision repair, suggesting active cell division. In contrast, M2 was enriched in many pathways associated with robust cell metabolism, including photosynthetic oxidative phosphorylation, amino acid and sugar biosynthesis, carbon fixation, nitrogen metabolism and sulfur metabolism, consistent with processes required for active leaf functions. M5 was enriched in several degradative pathways including fatty acid degradation, β-alanine metabolism, RNA transport and degradation, proteosome, lysosome, and ubiquitin-mediated proteolysis consistent with an expression profile associated with aging and senescence of leaves. M6 was not significantly enriched for any specific KEGG pathways. M10, which was similar in general expression profile to M2, was enriched for phenylpropanoid biosynthesis, amino sugar and nucleotide metabolism, pentose and glucuronate interconversions, steroid biosynthesis, and oxidative phosphorylation. M12 was not enriched significantly in any pathway, and M13 was significantly enriched only for protein processing in endoplasmic reticulum. Three pathways associated in some form with ribosomes were enriched in M14. Biosynthetic pathways including amino acid biosynthesis, pyrimidine metabolism, selenocompound metabolism, and porphyrin and chlorophyll metabolism were enriched in M15 where gene expression was higher at the earlier leaf harvest dates as compared to later harvest dates. Two apparently interrelated pathways, namely plant MAPK signaling and plant-pathogen interaction were significantly enriched in M16, which had an increase in expression of genes over time up to the last harvest date.

M3 and M4 contained genes that were expressed in similar profile, but at a higher level across all harvest dates in either Summer (M3) or Kanlow (M4) leaves, suggesting these genes could be cultivar specific, but these modules were not significantly enriched in any specific KEGG pathways. Similarly, M7 and M9 contained genes that were more abundantly expressed in Kanlow leaves (M7) or Summer leaves (M9). M7 was significantly enriched only in protein processing in endoplasmic reticulum pathways, and M9 was enriched tryptophan metabolism and diterpenoid metabolism pathways.

M8 which contained genes expressed at a higher level in Kanlow with increasing expression at each harvest date was significantly enriched in RNA transport, RNA degradation, and spliceosome suggesting cultivar differences in the modulation of these pathway genes. M11 was significantly enriched only in the carotenoid biosynthesis pathway.

### Expression profiles provided clues for differential expression and genomic presence of potential defense-related/environmental sensing genes in switchgrass populations

**Fig 6:**
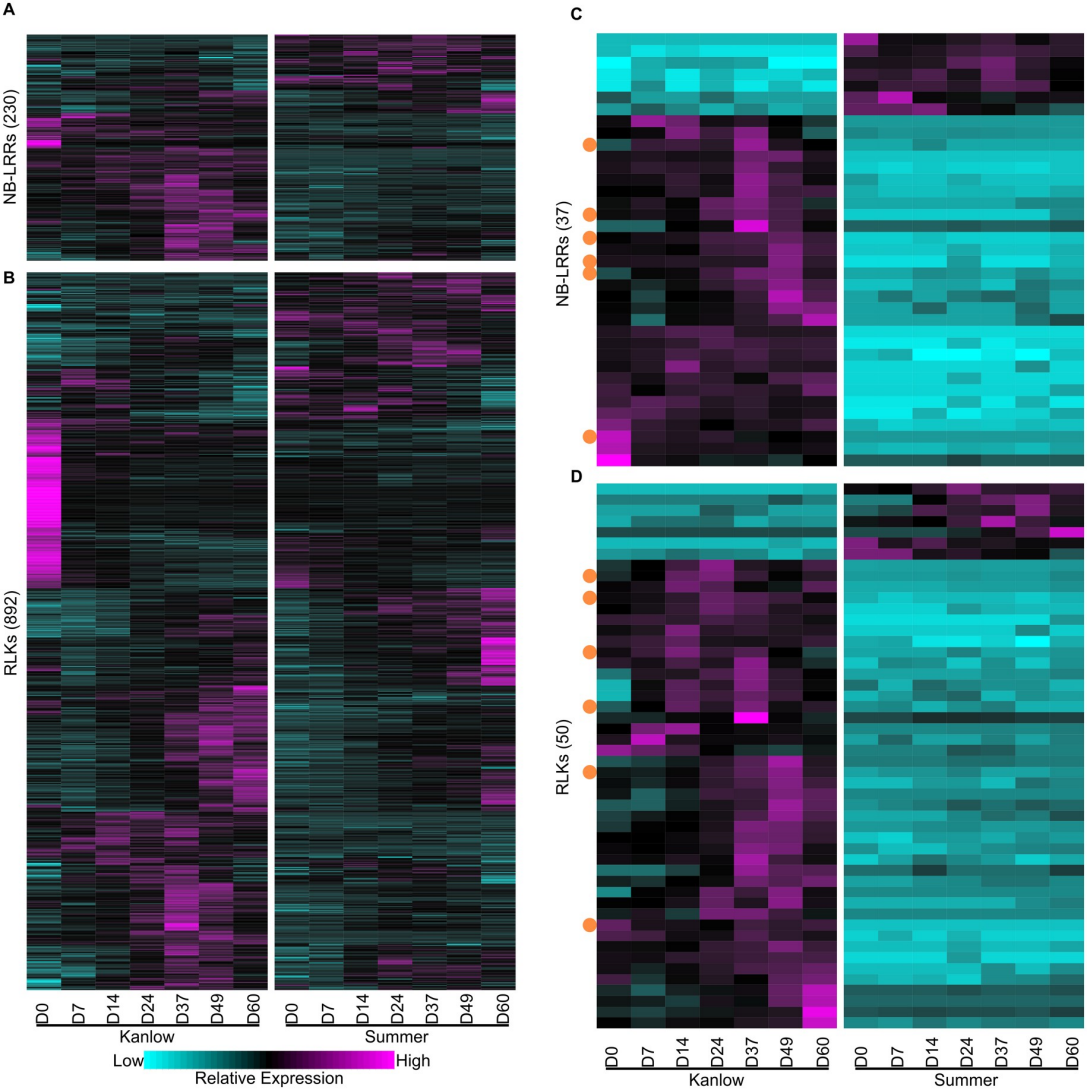
Expression profiles of potential defense-related genes. (A) Nucleotide-binding leucine-rich repeat (NB-LRRs) (B) Receptor-like kinases (RLKs), (C) NB-LRRs unique to or significantly more (log_2_-fold change ≥ 3) abundant in one or the other cultivar, (D) RLKs unique to or significantly more (log_2_-fold change ≥ 3) abundant in one or the other cultivar. For heat maps, one way clustering was performed and the dendrograms removed for ease of presentation Expression profiles are shown as z-scores with magenta = high expression and cyan = low/no expression. Orange dots in (C) and (D) indicate genes potentially not found in the Summer genome (‘upland absent’).

Expression of NB-LRRs and receptor-like kinases (RLKs), two large families of genes related to environmental sensing in plants were analyzed (Fig 6). Out the anticipated 1,011 NB-LRRs encoded in the switchgrass genome, expression evidence for 230 genes were detected (Fig 6A; S1 Data). Evidence indicated that clusters of genes were differentially expressed in each switchgrass cultivar, along with temporal differences in transcript abundance. Fifty-two NB-LRRs were more significantly expressed in Summer leaves than in Kanlow leaves. Conversely, 162 NB-LRRs were more abundantly expressed in Kanlow leaves. Peak expression for several NB-LRRs was seen at D37 and D49 for Kanlow leaves, with a few of them more highly expressed at the last harvest date (Fig 6A).

Expression evidence for a total of 892 RLKs was determined (Fig 6B). Similar to the expression profiles seen with NB-LRRs, there were both ecotypic and temporal variation in transcript abundances of RLKs. Notably, there was a significant abundance of transcripts in Kanlow leaves at the first harvest date (D0; Fig 6B) for several RLKs. These genes were subsequently downregulated. RLKs that had greater expression in Summer or Kanlow leaves were detected across several harvest dates, and many were most abundant in senescent leaves collected at the last harvest date (D60, Fig 6B).

Population-specific gene expression for both NB-LRRs and RLKs was also observed. These genes had a log_2_-fold change ≥ 3 between their transcript abundances and were significantly differentially expressed when comparing Kanlow and Summer leaves (S1 Data). Conceivably, such genes could be important to cultivar-specific differences in resistance to pathogens. A total of 37 NB-LRRs fit this category of cultivar specific expression (Fig 6C). Of these 37 NB-LRRs, seven were uniquely found to be expressed in Summer leaves, and 30 were uniquely expressed in Kanlow leaves (Fig 6C; S1 Data). Fifty RLKs fit the classification for cultivar-specific expression, with transcripts for seven genes found in Summer leaves and transcripts for 43 in Kanlow leaves (Fig 6D).

The presence or absence of defense-related genes could contribute to the documented differential responses of Kanlow and Summer plants to foliar pathogens. The cultivar specific expression patterns seen in Fig 6C and 6D could be caused by two factors: (1) cultivar (or potentially ecotype) specific expression networks (‘ecotype network’) or (2) a potential absence of the entire gene in the upland cultivar Summer (‘Summer absent’). Genomic resequencing data for both Kanlow and Summer were used to classify defense-related genes with Kanlow-specific expression into ‘ecotype network’ (genomic coverage across the gene coding region in both ecotypes, with singular expression in one ecotype) or ‘Summer absent’ (no genomic coverage across the gene coding region or detectable expression in Summer) types. Of the 30 NB-LRRs found expressed only in Kanlow leaves, six were classified as ‘Summer absent’ (orange dots, Fig 6C) and the remaining 24 as ‘ecotype network’. Similarly, of the 50 RLKs expressed only in Kanlow, six were also classified as ‘Summer absent’ (orange dots, Fig 6D) and the remaining 44 as ‘ecotype network’. It is plausible that a ‘Summer/upland-unique’ network exists, but the lack of full annotation of these genomes currently precludes its discovery.

**Fig 7:**
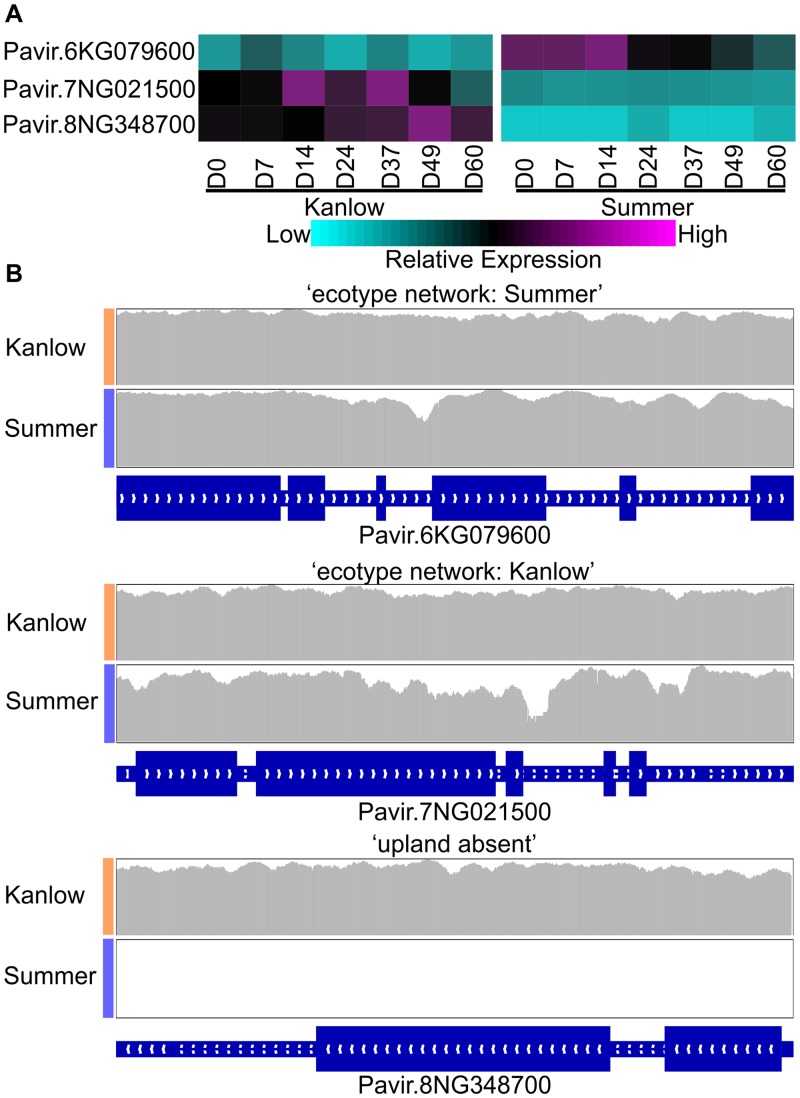
Expression heatmap and genomic coverage of three select NB-LRR genes. (A) Expression heatmap and (B) Genomic coverage evidence used for the ‘ecotype network’ or ‘Summer absent’ classification of NB-LRRs in Kanlow and Summer genomes. Gene models were obtained from Phytozome (https://phytozome.jgi.doe.gov/pz/portal.html#!info?alias=Org_Pvirgatum_er). Introns and exons are shown as narrow or wide rectangles respectively.

Examples of the ‘ecotype network’ and ‘Summer absent’ classifications are shown in Fig 7 for three NB-LRRs (Pavir.6KG079600, Pavir.7NG021500, and Pavir.8NG348700) taken from Fig 6C. Transcripts for Pavir.6KG079600 were significantly more abundant in Summer leaves than in Kanlow leaves, and transcripts for Pavir.7NG021500 were significantly enriched in Kanlow leaves than in Summer leaves (Fig 7A). Transcripts for Pavir.8NG358700 were only detected in Kanlow leaves (Fig 7A).

Coverage across gene coding regions derived from genomic resequencing for the three NB-LRRs are shown in Fig 7B. For Pavir.6KG079600 and Pavir.7NG021500, genomic coverage across the entire length of the predicted gene was similar in the two cultivars, resulting in ‘ecotype network’ classifications based on the expression data. In contrast, for Pavir.8NG348700, genomic coverage was only detected in Kanlow, suggesting that Pavir.8NG348700 was missing from the Summer genome and resulting in an ‘upland absent’ classification. Similar data (not shown) were observed for other ‘upland absent’ NB-LRR and RLK-encoding genes expressed only in Kanlow (orange dots, Fig 6C and 6D).

## Discussion

This study was undertaken to develop a foundation baseline of the transcriptomic changes occurring in leaves of two specific cultivars of switchgrass, Kanlow and Summer, grown under controlled conditions in a greenhouse. The expectation was that comparisons between the two cultivars could provide data on the commonalities and unique aspects of the transcriptomes during leaf development. The selection of these specific cultivars was because the lowland cultivar Kanlow and the upland cultivar Summer have been essential to the switchgrass breeding program of the Agricultural Research Service at Lincoln, Nebraska, USA [61]. A specific goal was to discover if genes important to sensing the environment were expressed in a similar or dissimilar in the two cultivars, because Kanlow and Summer have divergent response to plant foliar pathogens [17, 19]. Our data indicated that several NB-LRR and RLK genes were uniquely and significantly expressed in one or the other cultivar, and for some, genomic resequencing provided some evidence for the absence of specific genes. These underlying variations in gene expression and gene content could partially explain the differences in the divergent responses of the two cultivars to foliar pathogens.

Kanlow and Summer belong to different heterotic groups and both F_1_ and stabilized progeny between these cultivars show improved agronomic traits [10, 11, 42]. Selection of the 4^th^ leaf was based on a few observations: (1) in preliminary trials these leaves emerged at approximately the same times under the growth conditions of the greenhouse for both cultivars; (2) these leaves expanded and developed in a manner more comparable to leaves on field-grown plants; and (3) these leaves normally developed prior to the formation of secondary tillers.

Overall, both the phenotypic data on chlorophyll and N content were similar for leaves harvested from both cultivars. Leaf chlorophyll and N content are common markers for leaf senescence [38, 62]. Chlorophyll content increased over the first few harvests and decreased at the later harvest dates, indicating that the senescence program had been executed. Percent N decreased in the leaves from both cultivars with each successive harvest, indicative of both leaf expansion at the earlier harvest dates and potentially remobilization at the later harvest dates when leaf senescence had started. These data indicated that the general pattern of leaf development was similar for both cultivars, and there appeared to be no significant cultivar effects, at least for the 4^th^ leaf.

MDS analysis of the transcriptomic data supported the similarities in leaf development for both cultivars and indicated possible ecotypic signatures that differentiated them. A significant outlier appeared to be the first harvest of Kanlow leaves. A plausible reason could lie in the time of collection, when Summer leaves could have been at a transcriptionally different developmental time point as compared to Kanlow leaves. However, only minor differences in leaf chlorophyll and N content were observed between the two cultivars at this harvest date.

While there were some variations in the global gene expression profiles between the two cultivars, the expression of genes associated with normal leaf metabolism such as those associated with chloroplast functions, and C and N assimilation had largely similar profiles. These data corroborated previous transcriptomic findings obtained for flag leaves of field-grown Summer plants [38] and indicated that leaf development and physiology followed a similar trajectory in both Kanlow and Summer plants. An inherent assumption in these predicted physiological analyses was that switchgrass proteins with significant homology to well-characterized proteins in other plants function in a similar manner in switchgrass leaf metabolism. Relatively few switchgrass genes have been directly evaluated for function in-planta.

A recent report comparing switchgrass leaf transcriptomes collected at one harvest date suggested differential regulation of genes associated with C4-photsynthetic metabolism in upland as compared to lowland ecotypes [41]. Based on the experimental conditions outlined by Serba et al. [41], only one genotype was analyzed per ecotype, consisting of clonally propagated plants. Additionally, one leaf was harvested from each of three clones at one time point. RNA-Seq analyses of these samples could have maximized differences attributable to leaf developmental age, and genotype-specific variations in gene expression. To correct for these issues, we pooled samples from many plants at each sampling point to minimize genotypic variations in gene expression and attempted to more carefully match leaf age. Outside of the D0 Kanlow samples (discussed later), there were strong similarities across multiple measures of leaf physiology and development between the upland Summer and lowland Kanlow plants. Our more robust dataset did not uncover differential up/down regulation of genes associated with C4-photsynthetic metabolism, and more generally in several other aspects of leaf function, suggesting that multiple harvests would be needed to delineate any potential ecotype-linked variations in gene expression.

Similar to the profiles noticed for leaf functions, the onset and progression of leaf senescence appeared to be executed around the same developmental stage in both cultivars. A key marker for the onset of leaf senescence is the upregulation of genes encoding proteins required for chlorophyll catabolism [63]. For both Summer and Kanlow leaves, a noticeable upregulation of chlorophyll catabolic genes occurred between the D37 and D49 harvest dates. Changes in the expression of genes associated with chlorophyll catabolism were mirrored by changes in the expression of genes encoding proteins needed for β-oxidation and the glyoxylate cycle. Increased β-oxidation and glyoxylate cycle have been linked to leaf senescence in switchgrass flag leaves [38] and in other species [64].

In switchgrass, specific NAC and SAG genes have been linked to leaf senescence [38–40]. PvNAC1 was more abundantly expressed in the lowland Kanlow leaves and, conversely, both NAC29 and NAMB1 homologs were more abundantly expressed in Summer leaves. It has been postulated that the NAMB1 homologs could be involved in nutrient remobilization from switchgrass flag leaves [38] in a manner akin to those in wheat [65]. Variations in the expression of these NAC genes in Kanlow and Summer leaves could arise from subtleties in the execution of the leaf senescence program, the nature, types, and amounts of minerals and nutrients mobilized out from senescing leaves, or the result of experimental variables. Switchgrass populations differ in the amount of minerals and other nutrients that are remobilized from shoots to the below-ground portions of the plant over the course of the growing season [66, 67], indicating differences exist in end of season nutrient remobilization.

Although many putative switchgrass SAGs were expressed in a similar manner in both populations, variations in the expression of other SAG orthologs pointed to plausible differences during leaf senescence in Kanlow and Summer. As examples, in senescent Summer leaves there was a strong upregulation of a gene encoding a cytosolic NADP-malic enzyme, which could be involved in generating substrate level NADPH for senescence-related biosynthetic processes and/or maintaining cellular redox in the cytosol at a time when organelles were losing functions. Similarly, two genes encoding a catalase and lumazine synthase were strongly upregulated in senescing Summer leaves. Catalase has been implicated in cellular protection during leaf senescence [38, 68] and lumazine synthase has been implicated in the jasmonic acid pathway and senescence [69].

In Kanlow leaves, other genes were upregulated during senescence. Among these genes were an Arabidopsis WRKY6 ortholog involved in different aspects of metabolism and senescence [70, 71], and a putative ferulate 5-hydroxylase encoding gene that is required for efficient anthocyanin biosynthesis [72]. Increased anthocyanin biosynthesis has been linked to delayed leaf senescence in *Populus* [73], suggesting different mechanisms can contribute to protecting switchgrass leaves during the later stages of senescence to potentially permit an orderly resorption of nutrients from senescing leaves.

As expected, many functional aspects of leaf development and senescence were similar between Kanlow and Summer leaves, with some variations in the gene co-expression profile modules. Specifically, gene members of modules M3 and M4 where were more abundantly expressed in Summer leaves or in Kanlow leaves respectively. Although, no clear KEGG enrichment pathways were apparent in these modules, differences in the expression of genes encoding different classes of proteins were seen. How these different genes influence plant physiology are not currently known.

Significant dissimilarities in the expression profiles of genes linked to innate immunity and defense were observed between Kanlow and Summer leaves, providing a window into potential genetic determinants for their differential responses to pathogens [17, 19, 32]. Because mapping of transcripts was conducted on the available annotated genome that is based on the lowland tetraploid cultivar Alamo, which is more closely related to Kanlow than it is to Summer [74] it is possible that some transcripts present in Summer samples went unmapped. It is estimated that the upland and lowland switchgrass ecotypes diverged around 0.7–1 million years ago [75, 76] and several changes could have occurred at the genomic level, leading to loss/gain of genes and adaptations to their environment of origin [77]. Notably, genes encoding NB-LRRs and RLKs were enriched in a cultivar-specific manner in Kanlow as compared to Summer. Query of available genomic resequencing data indicated that beyond the evidence for the lack of expression of some of these NB-LRR and RLK genes, there was also an apparent absence of other NB-LRRs and RLKs in the Summer genome. Whether these genes directly impact differential pathogen resistance documented for the two cultivars is not known yet, but they suggest that Kanlow plants may have a more robust mechanism to monitor and respond to biotic stressors. It is plausible that these ecotypic and/or cultivar-specific genes and gene-networks could be the driver for the documented differences in responses to foliar pathogens between these two cultivars. Incorporating broad resistance to plant pathogens in elite switchgrass germplasm that combine Kanlow and Summer genetics [78] could further improve sustainability of biomass production under low-input conditions.

Future studies will include cultivar-dependent transcriptional responses to foliar pathogens, and evaluation of progeny derived by intermating select Kanlow and Summer plants under field and greenhouse conditions for pathogen-resistance and host responses. We also anticipate that combining genotyping with phenotyping will yield suitable genetic markers that can guide breeding efforts. Much of this combined information gleaned from switchgrass should be applicable to the study of related perennial grasses.

## Supporting information

S1 Data Gene annotations and module memberships. (XLSX)
